# Comparative study of Exosome Ovarian Cancer auxiliary diagnostic kit (Chemiluminescence) and Serum Tumor Marker CA125 in the diagnosis of Ovarian Epithelial Cell Carcinoma

**DOI:** 10.12669/pjms.41.11.12315

**Published:** 2025-11

**Authors:** Wei Lu, Chunli Yang, Tian Liu, Lichao Guo, Chengbi Tong

**Affiliations:** 1 Wei Lu, Department of Laboratory Pathology, The Hospital of 82nd Group Army PLA, Baoding 071000, Hebei, China; 2 Chunli Yang, Department of Laboratory Pathology, The Hospital of 82nd Group Army PLA, Baoding 071000, Hebei, China; 3 Tian Liu, Department of Laboratory Pathology, The Hospital of 82nd Group Army PLA, Baoding 071000, Hebei, China; 4 Lichao Guo, Department of Laboratory Pathology, The Hospital of 82nd Group Army PLA, Baoding 071000, Hebei, China; 5 Chengbi Tong Clinical Laboratory, Affiliated Hospital of Hebei University, Baoding 071000, Hebei, China

**Keywords:** Exosomal Ovarian Cancer Kit, CA125, Epithelial Ovarian Cancer, Diagnostic

## Abstract

**Objective:**

To compare the diagnostic performance of an exosomal ovarian cancer assay (chemiluminescence) with serum CA125 for epithelial ovarian cancer (EOC), focusing on sensitivity, specificity, positive predictive value (PPV), and negative predictive value (NPV).

**Methodology:**

This retrospective study included 200 patients from the Department of Obstetrics and Gynecology, Hospital of 82nd Group Army PLA (June 2023 to December 2024). Participants were divided into a control group (n = 150, including healthy individuals, patients with pelvic inflammatory disease, and other tumors) and an EOC group (n = 50, confirmed by pathology). Serum samples were analyzed using the exosomal kit and CA125. Agreement metrics (PPA, NPA, OPA) with 95% CIs and Kappa consistency were calculated.

**Results:**

There were significant differences in age distribution and menopausal status between the two groups (*P*<0.05), with marked differences also found in serum HE4, serum CA125, and OCS values between the two groups (*P*<0.05). The exosomal assay showed a sensitivity of 74.0%, specificity of 99.3%, PPV of 97.4%, and NPV of 92.0%, outperforming CA125 (sensitivity 40.0%, specificity 97.3%, PPV 83.3%, NPV 83.0%). Kappa analysis indicated good agreement between methods (K = 0.797, P < 0.01).

**Conclusion:**

The exosomal ovarian cancer diagnostic kit exhibits superior sensitivity and specificity compared to CA125, suggesting strong potential as a complementary tool for early EOC detection.

## INTRODUCTION

Ovarian cancer is the deadliest malignant tumor of the female reproductive system, with a five-year survival consistently hovering around a low rate of 38.8%.^1^ Epithelial ovarian cancer (EOC) is the main pathological type, accounting for over 90% of malignant ovarian tumors,^2^ and its diagnosis continues to face significant challenges. Due to the deep location of the ovaries and the lack of specific symptoms, approximately 70% of patients are diagnosed at an advanced stage (III-IV), resulting in poor prognosis.

Current clinical diagnostics rely on imaging and serum tumor markers. Although transvaginal ultrasound can detect structural abnormalities, its sensitivity for distinguishing benign from malignant conditions is only 74-83%.^3^ The most widely used biomarker, cancer antigen 125 (CA125), suffers from inadequate sensitivity and specificity.^4^ Its sensitivity is particularly low for early-stage lesions (around 50%) and certain subtypes such as mucinous and clear cell carcinomas.^5^ It is also elevated in benign conditions like endometriosis and pelvic inflammatory disease, leading to a high false-positive rate.^6^

Given the limitations of CA125, there is an urgent clinical need for more accurate diagnostic tools. Exosome-based assays have emerged as a promising novel approach. Therefore, the primary objective of this study was to perform a head-to-head comparison between a novel chemiluminescence-based exosomal ovarian cancer auxiliary diagnostic kit (OCS) and the conventional serum CA125 test, to evaluate whether this new technology offers superior sensitivity and specificity for diagnosing EOC.

Exosomes (30-150 nm extracellular vesicles) have recently gained attention as potential biomarkers for tumor diagnostics. They carry tumor-specific proteins, lipids, and nucleic acids, offering novel insights for precision medicine. For instance, exosomal HSP70 and CD63 are highly expressed in ovarian cancer,^7^,^8^ and miRNAs such as miR-373 and miR-205 are significantly upregulated in patient-derived exosomes.^9^,^10^ Notably, detecting CA125 within exosomes has shown superior sensitivity to traditional serum assays.^11^ Recently, China approved the first exosomal ovarian cancer diagnostic kit, demonstrating high sensitivity (95.4%) and specificity (90.4%).^12^ In this study, we utilized an exosomal ovarian cancer auxiliary diagnostic kit to comprehensively compare key diagnostic parameters with the conventional CA125-based system.

## METHODOLOGY

This was a retrospective, cross-sectional diagnostic accuracy study with no longitudinal follow-up component. All analyses were conducted on retrospectively collected data obtained at the time of patient enrollment. A total of two hundred patients who visited the Department of Obstetrics and Gynecology at the Hospital of 82nd Group Army PLA from June 2023 to December 2024 were selected as the study subjects and were divided into an interference group (n=150, including 40 healthy individuals, 55 with gynecological pelvic inflammatory disease, and 55 with other types of tumor) and a test group (n=50, pathologically confirmed as having epithelial ovarian cancer postoperatively).

### Ethical Approval:

The study was approved by the Institutional Ethics Committee of the Hospital of 82nd Group Army PLA (No.:2023002; Date: March 23, 2023), and written informed consent was obtained from all participants.

### Inclusion criteria:

- Female patients aged ≥ 18 years.
- Clinically diagnosed or pathologically confirmed as suffering from epithelial ovarian cancer.
- Clinically diagnosed with benign gynecological diseases (e.g., pelvic inflammatory disease, uterine fibroids, benign ovarian cysts).
- Healthy individuals without obvious gynecological disease symptoms.

### Exclusion criteria:

- Patients with other malignant tumors or a history of malignancy.
- Those with severe heart, liver, or kidney dysfunction or other systemic diseases.
- Those who had undergone ovarian-related surgery or chemotherapy.
- Those who refused to participate in the study or had incomplete data.

Clinical data were collected from all patients, including age, menstrual status, and test results of serum CA125 and serum HE4. The average age of the test and interference groups was (61.66±13.41) and (44.08±14.19) years, respectively. Specifically, the test group included 43 postmenopausal and seven premenopausal patients, with 53 postmenopausal and 97 premenopausal patients in the interference group.

### Diagnostic Criteria:

Diagnostic criteria for serum CA125, serum HE4, and exosomal OCS. Following the “Expert Consensus on the Application of Gynecological Tumor Markers”,^1^ the critical value for serum CA125 was set at 35 U/ml in this experiment. For premenopausal and postmenopausal patients, the critical value for serum HE4 was 68.96 pmol/L and 114.9 pmol/L, respectively, and results below or equal to these critical values were defined as negative, with those above being positive. The diagnostic criteria for exosomal OCS: positive if OCS > 0.506 and negative if OCS ≤ 0.506.

### Sample Collection and Processing:

5 ml of peripheral venous blood was collected from all patients at enrollment, and serum was separated by centrifuge at 3,000 rpm for 10 minutes after standing for 30 minutes, with a serum sample volume of no less than 1.0 ml. Separated serum samples were stored at 2-8°C for no more than 24 hours and at below -20°C for no more than three months, with a maximum of three freeze-thaw cycles. Frozen samples were thawed at room temperature (no repeated freezing and thawing) and mixed well for later use. In case of any floccules in the samples, the supernatant should be centrifuged before use.

### Detection Methods:

Exosomal Ovarian Cancer Auxiliary Diagnostic Kit (Chemiluminescence): The kit used for exosomal detection was provided by Shanghai 3D Biomedicine Science & Technology Co., Ltd., operated following the instructions to detect the expression levels of exosome-related markers in serum. Serum CA125 Detection: The chemiluminescent immunoassay (CLIA) was utilized, employing routine testing equipment and reagents from the clinical laboratory of the Hospital of 82nd Group Army PLA.

### Observation Indicators:

- *General Data:* Age distribution and the ratio of patients with menopause.
- *Serum Marker Levels:* Serum HE4, serum CA125, and OCS values.
- *Diagnostic Efficacy Indicators:* The diagnostic performance of exosomal OCS values, serum CA125, and serum HE4 in epithelial ovarian cancer is evaluated. The sensitivity (SE), specificity (SP), positive predictive value (PPV), and negative predictive value (NPV) for each indicator are calculated. The quantitative assessment of diagnostic efficacy is performed using the Receiver Operating Characteristic (ROC) curve, and the diagnostic accuracy is systematically evaluated by calculating the area under the curve (AUC) and its 95% confidence interval (95% CI) for each biomarker. Specifically, the AUC value ranges from 0.5-1.0, with the value approaching one indicating better diagnostic efficacy. The consistency level of diagnostic results with the gold standard was assessed using Cohen’s Kappa coefficient, in which Kappa values were set from -1 to 1, with a Kappa value > 0.8 for very strong consistency, 0.6-0.8 for significant consistency, and < 0.4 for poor consistency.

### Statistical analysis:

SPSS 27.0 software was used for the statistical analysis of data. Measurement data following the normal distribution were expressed as Mean ± SD, with inter-group differences compared using the t-test. Measurement data not following the normal distribution were expressed as median (upper and lower quartiles), with inter-group differences compared using the rank-sum test. Categorical sample sizes were expressed as percentages (%), with inter-group differences compared using the chi-square test of contingency tables. Following pathological results as the standard, ROC curves were utilized to assess the impact of each indicator on diagnosis, with SE, SP, NPV, PPV, AUC, and 95% CI calculated for each indicator. Additionally, pairwise comparisons of the sensitivity and specificity of each indicator were conducted using McNemar’s test. *P*<0.05 was considered statistically significant, and Kappa values were calculated to describe the consistency of diagnostic criteria.

## RESULTS

The analysis of the general data showed significant differences in the age distribution and the proportion of menopausal patients between the test and interference groups (*P*<0.05), as shown in Table-I.

**Table-I:** Analysis of General Data.

Indicator	Test Group (n=50)	Interference Group (n=150)	t/χ^2^	P
Age	61.66±13.41	44.08±14.19	7.909	<0.001
***Menopause***				
Yes	43(86.00%)	53(35.33%)	36.565	<0.001
No	7(14.00%)	97(64.67%)		

The histological subtypes of the 50 confirmed epithelial ovarian cancer cases are detailed in Table-II. The cohort was predominantly composed of high-grade serous carcinoma (76.0%), which is representative of the typical distribution of EOC subtypes.

**Table-II:** Histological Classification of Epithelial Ovarian Cancer Cases in the Test Group.

Histological Subtype	Number of Cases (n)	Percentage (%)
High-Grade Serous Carcinoma (HGSC)	38	76.0
Endometrioid Carcinoma	5	10.0
Clear Cell Carcinoma	4	8.0
Mucinous Carcinoma	2	4.0
Low-Grade Serous Carcinoma (LGSC)	1	2.0
Total	50	100

Comparison of the levels of serum HE4, serum CA125, and OCS values suggest substantial differences in serum HE4, serum CA125, and OCS values between the test and interference groups (*P*<0.05) Table-III.

**Table-III:** Comparison of Serum HE4, Serum CA125, and OCS Values.

Indicator	Test Group(n=50)	Interference Group (n=150)	z/χ^2^	P
Serum CA125	24.5(8.26,91.53)	11.75(8.15,16.1)	-3.600	<0.001
Serum HE4	165(62.27,369.25)	59.75(34.51,81.42)	-6.320	<0.001
OCS	0.71(0.5,0.89)	0.15(0.08,0.23)	-8.703	<0.001

**Fig.1:**
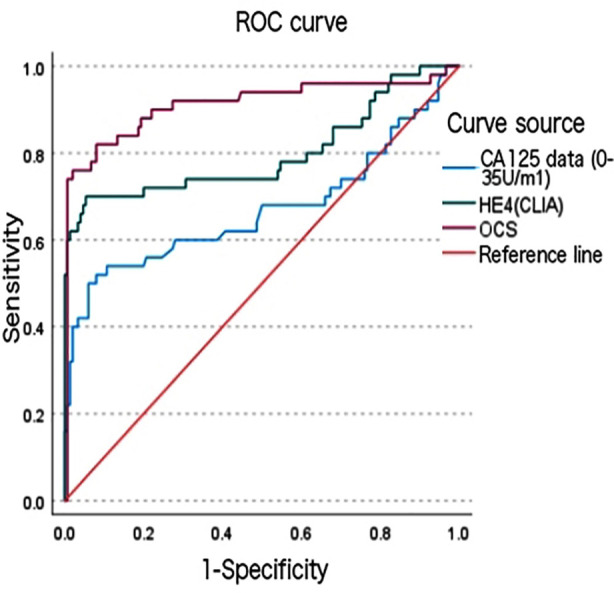
ROC Curve of Each Indicator.

Following the criteria of “positive if CA125 value > 35 U/ml, negative if premenopausal serum HE4 > 68.96 pmol/L, positive if postmenopausal serum HE4 > 114.9 pmol/L, and positive if OCS value > 0.506 being”, the SE, SP, PPV, and NPV for each indicator were calculated. Meanwhile, ROC curves were plotted to derive the AUC and 95% CI. The test results (Table-IV and Fig.1) showed that the AUC for OCS (0.911, 95% CI: 0.851-0.971) was significantly higher than that for serum CA125 (0.670, 95% CI: 0.565-0.775) and serum HE4 (0.799, 95% CI: 0.709-0.888), indicating the optimal overall diagnostic efficacy for OCS. In terms of sensitivity, OCS (0.740) was close to serum HE4 (0.700) but higher than serum CA125 (0.400), suggesting the highest risk of missed diagnosis with CA125. As for specificity, both OCS (0.993) and serum CA125 (0.973) performed excellently, surpassing serum HE4 (0.747), indicating a higher risk of misjudgment of negative samples with HE4. In terms of PPV, OCS (0.974) was far superior to serum CA125 (0.833) and HE4 (0.479), indicating that OCS boasts the highest reliability for positive results, while HE4 faces significant false positive issues. For NPV, OCS (0.920) was close to HE4 (0.882) and both were superior to CA125 (0.830), suggesting strong exclusion capabilities for negative samples with OCS and HE4. Meanwhile, the Kappa coefficient indicated that the diagnostic consistency of OCS (0.797) was significantly higher than that of serum CA125 (0.452) and HE4 (0.387), with the latter two failing to achieve ideal consistency levels. In conclusion, OCS was generally superior in terms of AUC, specificity, PPV, and consistency, making it the best overall indicator; serum HE4 had sensitivity close to OCS but lacked specificity and had a low PPV; and serum CA125 had comparable specificity to OCS but severely lacked sensitivity, thereby requiring cautious consideration for its clinical applications.

**Table-IV:** Diagnostic Efficacy of Serum HE4, Serum CA125 and OCS Values.

Indicator	AUC value	P	SE	SP	PPV	NPV	Kappa
OCS	0.911(0.851-0.971)	<0.001	0.740	0.993	0.974	0.920	0.797
Serum CA125	0.670(0.565-0.775)*	0.002	0.400	0.973	0.833	0.830	0.452
Serum HE4	0.799(0.709-0.888)*	<0.001	0.700	0.747	0.479	0.882	0.387

***Note:*** Compared to OCS test,

* *P*<0.05

To investigate the potential significant differences in sensitivity and specificity among various diagnostic indicators, McNemar’s test was further employed for pairwise comparison analyses of the diagnostic indicators. The results indicated (Table-V) the specificity and sensitivity of the indicators as follows: OCS > serum HE4 (c^2^=25.92, *P*<0.001) > CA125 (c^2^=7.348, *P*=0.007). According to the above diagnostic efficacy results, OCS outperformed serum CA125 and serum HE4 in diagnosing epithelial ovarian cancer.

**Table-V:** McNemar’s Test Results for HE4, CA125 and OCS Values.

Indicator	χ^2^	P
OCS vs SerumHE4	24.02	<0.001
OCS vs Serum CA125	8.167	0.004
Serum HE4 vs Serum CA125	40.695	<0.001

## DISCUSSION

This study demonstrates that the exosomal OCS assay significantly outperforms conventional serum CA125 and HE4 tests, showing markedly higher sensitivity (74.0% vs. 40.0% and 70.0%, respectively) and superior overall diagnostic accuracy (AUC 0.911 vs. 0.670 and 0.799). These findings align with recent literature on exosome-based diagnostics. For instance, Chen et al.^11^ reported that exosomal CA125 detection demonstrated superior sensitivity compared to traditional serum assays, consistent with our observations. Additionally, the recent approval of China’s first exosomal ovarian cancer diagnostic kit in 2024 further supports the clinical potential of this approach.^12^

Our results showing OCS’s superior performance are particularly noteworthy given the well-documented limitations of CA125.^5^,^6^ The low sensitivity of CA125 in early-stage disease and specific histological subtypes has been extensively reported,^13^,^14^ and our findings confirm these limitations while demonstrating how exosomal biomarkers may overcome them. The higher AUC value of OCS (0.911) compared to both conventional biomarkers aligns with previous studies suggesting that exosome-based approaches provide more accurate diagnostic information.^15^,^16^ The excellent specificity maintained by OCS (99.3%) while achieving substantially improved sensitivity addresses a critical challenge in ovarian cancer diagnostics. This balance between sensitivity and specificity has been a persistent obstacle in the field, and our results suggest that exosome-based assays may offer a solution. The strong diagnostic consistency indicated by the Kappa value (0.797) further supports the reliability of the OCS assay.

When compared with other emerging biomarkers, our findings suggest that OCS performs favorably.^8^ For example, while HE4 has shown promise as an alternative to CA125,^17^,^18^ our direct comparison demonstrates clear advantages of the exosomal approach. The multi-analyte nature of the OCS assay likely contributes to its superior performance, as it integrates various biological information rather than relying on a single biomarker.^7^,^19^

### Limitations:

First, its retrospective design and relatively limited sample size may introduce potential selection biases and affect the generalizability of the findings. Therefore, the conclusions require validation in larger, more diverse, and prospective patient populations to ensure universality and reliability. Second, despite the outstanding performance of OCS, the use of a single indicator for diagnosis often comes with limitations in practical clinical applications. The model of combined diagnosis involving multiple indicators should be further explored to enhance the diagnostic accuracy of epithelial ovarian cancer. Furthermore, diagnostic criteria should be refined for patients of different ages and menopausal statuses to better meet clinical diagnostic needs.

## CONCLUSIONS

This study has demonstrated that OCS outperforms serum CA125 and serum HE4 in diagnosing epithelial ovarian cancer through an in-depth analysis of these three indicators, which provides new ideas and directions for the diagnosis of epithelial ovarian cancer.

### Authors’ Contributions:

**WL** and **CY:** Designed this study, prepared this manuscript, are responsible and accountable for the accuracy or integrity of the work.

**TL** and **LG:** Collected and analyzed clinical data. Critical Review.

**CT:** Significantly revised this manuscript.

All authors have contributed to the writing of the manuscript, read and approved the final version.
